# Phase Equilibria, Crystal Structure and Hydriding/Dehydriding Mechanism of Nd_4_Mg_80_Ni_8_ Compound

**DOI:** 10.1038/srep15385

**Published:** 2015-10-16

**Authors:** Qun Luo, Qin-Fen Gu, Jie-Yu Zhang, Shuang-Lin Chen, Kuo-Chih Chou, Qian Li

**Affiliations:** 1 State Key Laboratory of Advanced Special Steels, Shanghai University, Shanghai 200072, China; 2 Australian Synchrotron, 800 Blackburn Rd, Clayton 3168, Australia; 3 Institute of Genomic Material, Shanghai University, Shanghai 200444, China; 4 CompuTherm, LLC, Madison, WI 53719, USA

## Abstract

In order to find out the optimal composition of novel Nd-Mg-Ni alloys for hydrogen storage, the isothermal section of Nd-Mg-Ni system at 400 °C is established by examining the equilibrated alloys. A new ternary compound Nd_4_Mg_80_Ni_8_ is discovered in the Mg-rich corner. It has the crystal structure of space group *I*4_1_/*amd* with lattice parameters of *a* *=* *b* = 11.2743(1) Å and *c* = 15.9170(2) Å, characterized by the synchrotron powder X-ray diffraction (SR-PXRD). High-resolution transmission electron microscopy (HR-TEM) is used to investigate the microstructure of Nd_4_Mg_80_Ni_8_ and its hydrogen-induced microstructure evolution. The hydrogenation leads to Nd_4_Mg_80_Ni_8_ decomposing into NdH_2.61_-MgH_2_-Mg_2_NiH_0.3_ nanocomposites, where the high density phase boundaries provide a great deal of hydrogen atoms diffusion channels and nucleation sites of hydrides, which greatly enhances the hydriding/dehydriding (H/D) properties. The Nd_4_Mg_80_Ni_8_ exhibits a good cycle ability. The kinetic mechanisms of H/D reactions are studied by Real Physical Picture (RPP) model. The rate controlling steps are diffusion for hydriding reaction in the temperature range of 100 ~ 350 °C and surface penetration for dehydriding reaction at 291 ~ 347 °C. *In*-*situ* SR-PXRD results reveal the phase transformations of Mg to MgH_2_ and Mg_2_Ni to Mg_2_NiH_4_ as functions of hydrogen pressure and hydriding time.

Solid-state hydrogen storage was considered as the safest and most effective way to use the H_2_ as prominent energy carrier in the future^1^. Magnesium-based alloys have been extensively investigated as potential materials for solid-state hydrogen storage due to its reasonably high hydrogen capacity (7.6wt.%) and could be interesting if it is considered as a heat storage material in heat power plants^2^. However, the relatively slow kinetics of H/D reaction makes them far from practical application. Extensive efforts have been made to improve the H/D properties of magnesium-based alloys by adopting novel preparation techniques^3^,^4^ to reduce the particles sizes or/and adding catalytic components such as transition metals (Ni^3^, Nb^5^, Ti^6^) and rare earth elements (RE = La^7^, Ce^8^, Pr^9^, Nd^10^). The addition of RE into Mg-based hydrogen storage alloys facilitates hydrogen absorption through the formation of rare earth hydrides (REH_x_). The REH_x_ act as active nucleation sites for magnesium hydride by chemisorbing hydrogen atoms and transferring them to the Mg-metal interfaces^7^,^11^. Among the catalytic elements, Nd shows good catalytic effect on the H/D kinetics of Mg-Ni alloys, especially for the samples of ultrafine crystalline and small particle size^10^,^12^,^13^,^14^,^15^,^16^,^17^,^18^,^19^. Tanaka *et al.*^12^ found that the nanocrystallized Nd-Mg-Ni alloys prepared through melting-spinning followed by crystallizing exhibited excellent hydrogen absorbing kinetics and Pressure-Composition-Temperature (PCT) characteristics in comparison with those of the corresponding as-cast alloys with coarse eutectic structures. However, the growth up of the grain size would impair the hydrogen storage performance. Denys *et al.*^20^ found that the kinetic properties and cyclic stabilities of nanostructured Mg-based and Mg-8wt.%Mm-20wt.%Ni based hydrides degraded due to the grain growth of Mg nanocrystalline during thermal desorption above 300 °C. Therefore, it is important to keep the grain sizes of NdH_x_, Mg and Mg_2_Ni/Ni small as long as possible.

Zhu *et al.*^21^,^22^,^23^ reported the method of *in*-*situ* formation of CeH_2.73_-MgH_2_-Ni and YH_3_-MgH_2_-Mg_2_NiH_4_ nanocomposites through directly hydriding the Mg_80_Ce_18_Ni_2_ and Mg_12_YNi alloys. They found that the composites of CeH_2.73_-MgH_2_-Ni remained its excellent performance after 500 H/D cycles because the *in*-*situ* formed nanocomposite structure suppressed the grain growth of Mg and MgH_2_^21^. However, the as-melted Mg_80_Ce_18_Ni_2_ alloy was a multiphase mixture which was composed of 57 wt.% CeMg_3_, 29 wt.% Ce_2_Mg_17_, 7 wt.% CeMg, and 5wt.% CeMgNi_4_^21^. That is to say, the catalytic elements Ce and Ni didn’t distribute uniformly in the alloy, which would lead to the non-uniformity of *in*-*situ* formed CeH_2.73_-MgH_2_-Ni composites. It is reasonable to assume that if the catalytic elements distribute uniformly in the whole alloy rather than concentrate in a few phases, the *in*-*situ* formed nanocomposites will become more homogenous. One of homogenous alloys is amorphous. Nanocrystallisation of the amorphous LaMg_11_Ni alloy led to the formation of Mg_2_Ni/Mg_1.9_La_0.1_Ni and La_1.8_Mg_17_Ni_1_ nanocrystalline, which greatly improved the hydrogenation rate and lowered the temperature of hydrogen desorption^24^,^25^. Another easily available and stable alloy with homogenous microstructure is the single phase or intermetallic compound. Therefore, searching for the Mg-based multicomponent compound and synthesizing it with catalyst elements Nd and Ni are significant for *in*-*situ* formation of ultrafine NdH_x_-MgH_2_-Ni/Mg_2_NiH_4_ composites with excellent hydrogen storage properties. However, seeking for the Mg-based multicomponent compounds with good hydrogen storage properties is time-consuming through traditional trial-and-error method. A precise Nd-Mg-Ni phase diagram could help us to find a suitable target alloy for hydrogen storage and to select the reasonable process parameters for preparing it. The method combining the computational materials with the experimental verification would save a lot of time to explore new material.

Therefore, the purpose of this study is that how to explore the Nd-Mg-Ni hydrogen storage alloy with excellent properties for meeting the practical application by material design method in order to avoid the drawback of trial-and-error method, then the target alloy is prepared to verify validity. Moreover, the relationships between the thermodynamic property, kinetic performance and microstructure are systematically investigated in order to clarify the H/D mechanism. Specifically, through the first hydrogen-induced decomposition of the new ternary compound Nd_4_Mg_80_Ni_8_, the nanocomposites of NdH_2.61_-MgH_2_-Mg_2_NiH_0.3_ are *in*-*situ* formed. This alloy shows fast H/D rates and good cycling behavior. The mechanism of hydrogen-induced decomposition and phase evolution during hydriding reaction are systematically analyzed by HR-TEM and *in*-*situ* SR-PXRD. Moreover, the kinetics mechanism is theoretically investigated by RPP model to clarify our experimental results.

## Results and Discussions

### The phase diagram of Nd-Mg-Ni system in Mg-rich corner at 400 °C

**Figure 1:**
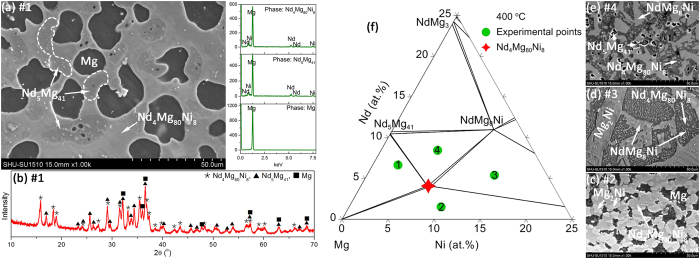
(**a**–**e**) The SEM images and XRD pattern of samples and (**f**) the isothermal section of Nd-Mg-Ni system in the Mg-rich corner at 400 °C.

The phase equilibria at 400 °C were determined by analyzing the phase composition of the four quenched samples. The XRD and EDS analysis of the alloys are listed in Table S1. In the Samples #1 and #2 annealed at 400 °C, an unknown phase (named as Nd_4_Mg_80_Ni_8_) is observed with an average composition of at.% 4.86Nd-87.46Mg-7.68Ni, which is different from Nd_5_Mg_41_, Mg, Mg_2_Ni and any other known ternary compounds. The morphology and XRD patterns of Sample #1 shown in Fig. 1(a,b) confirm the existence of the new ternary compound. The SEM images of Samples #2~4 in Fig. 1(c~e) show that the Nd_4_Mg_80_Ni_8_ equilibrates with Mg, Mg_2_Ni, NdMg_8_Ni and Nd_5_Mg_41_ phases. According to the experimental results, the determined phase diagram in the Mg-rich corner at 400 °C is plotted in Fig. 1(f).

The Nd-Mg-Ni ternary compounds are expected to be the hydrogen storage alloys with excellent properties because the uniform composition would leads to *in*-*situ* formation of the ultrafine MgH_2_-NdH_x_-Ni/Mg_2_NiH_4_ composites^11^,^21^,^26^. From the reported literature and our experimental work, it can be found that there are seven ternary compounds in the Nd-Mg-Ni system: Nd_4_Mg_80_Ni_8_, NdMg_8_Ni, NdMg_5_Ni^27^, NdMg_2_Ni^9^, Nd_2_MgNi_2_^28^, NdMgNi_4_^29^ and NdMg_2_Ni_9_^30^. The new compound Nd_4_Mg_80_Ni_8_ is selected as the target alloy because of its highest content of Mg among those ternary compounds which indicates the maximum hydrogen capacity.

### The crystal structure of Nd_4_Mg_80_Ni_8_

**Figure 2:**
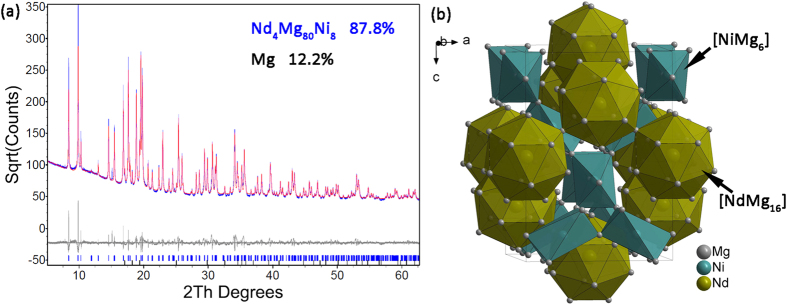
(**a**) The experimental (blue), fitted (red), and difference (grey line below observed and calculated patterns) SR-PXRD profiles for Sample #5 at a wavelength of 0.8262 Å and (**b**) the schematic crystal structure of Nd_4_Mg_80_Ni_8._

In order to determine the crystal structure of Nd_4_Mg_80_Ni_8_, the single phase was synthesized and examined by SR-PXRD. The actual composition of Sample #5 and #6 are Nd_4.08_Mg_89.98_Ni_5.94_ and Nd_4.45_Mg_84.64_Ni_10.91_, respectively. The SR-PXRD pattern of Sample #5 was indexed with a tetragonal unit cell using DICVOL06^31^. The structure solution started using the charge-flipping algorithm implemented in the program TOPAS v4.2^32^. The Ni and Nd atoms were easily located in the electron density maps. The structure was subsequently solved in the space group of *I*4_1_/*amd* (No. 141) by global optimization in direct space with 5 Mg atoms with no constraint using the program FOX^33^. Rietveld refinement was performed using TOPAS v4.2, and the refined lattice parameters were *a* = *b* = 11.2743(1) Å, *c* = 15.9170(2) Å, V = 2023.19(4) Å^3^. The diffraction profile fitted by Rietveld refinement using these parameters is shown in Fig. 2(a), with the agreement factors of *R*_wp_ = 8.1%, *R*_B_ = 6.4%, and *Go*F = 1.44. The fitting result suggests that there were 87.8 wt.% Nd_4_Mg_80_Ni_8_ and 12.2 wt.% Mg in the Sample #5. The details of the structure determination and crystallographic data are presented in Tables 1 and 2. The crystal structure of Nd_4_Mg_80_Ni_8_ is shown in Fig. 2(b).

Nd_4_Mg_80_Ni_8_ has a distinguished structure from other reported M-Mg-Ni (M = metal) ternary alloys. There is one symmetry independent Ni atom in the unit cell coordinates with six Mg atoms forming [NiMg_6_] in trigonal antiprism shape. Two different Ni-Nd bond distances are observed at 2.8186 and 2.8218 Å, which are close to the sum of the metallic radii *r*_Ni_ + *r*_Mg_ = 2.85 Å and larger than the sum of the covalent radii *r*_Ni_ + *r*_Mg_ = 2.75 Å^27^. While Nd atom in the unit cell coordinates with sixteen Mg atoms forming [NdMg_16_], where Nd atom sits in the middle of tetrahexahedron with the Nd-Mg bond distances equaling to 3.4501, 3.5464 and 3.5660 Å. The shortest bond distance is slightly larger than the sum of the metallic radii 3.42 Å. The trigonal antiprisms of [NiMg_6_] are linked together through vertices and the complex polyhedra of [NdMg_16_] also linked together via shared vertices. Figure 2(b) shows both [NiMg_6_] and [NdMg_16_] complexes form two independent three-dimensional network in the structure.

### The phase transformation of Nd_4_Mg_80_Ni_8_ during as-cast → anneal → hydrogenation process

**Figure 3:**
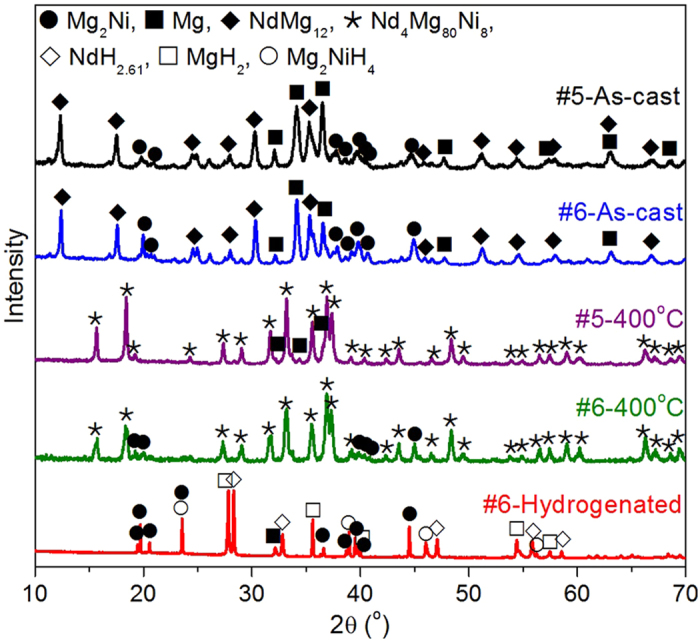
The XRD patterns of Nd_4_Mg_80_Ni_8_ with different heat treatment conditions.

The XRD patterns of as-cast Sample #5 and #6 shown in Fig. 3 show that both of the samples are multiphase alloys containing NdMg_12_, Mg and Mg_2_Ni. NdMg_12_ is a metastable phase in Nd-Mg system which forms if a nucleation barrier for Nd_5_Mg_41_ exists^34^. Therefore, the as-cast sample is not suitable for hydrogenation to *in*-*situ* form uniform nanocomposites of hydrides. According the established phase diagram, the equilibrated phase should be 90.7 wt.% Nd_4_Mg_80_Ni_8_ with 9.3 wt.% Mg in Sample #5 and 88.3 wt.% Nd_4_Mg_80_Ni_8_ with 11.7 wt.% Mg_2_Ni in Sample #6 at 400 °C. In order to obtain a homogeneous alloy, both alloys were annealed at 400 °C for 2 days. It is found that NdMg_12_ disappeared in the annealed samples and supplanted by Nd_4_Mg_80_Ni_8_. The predicted phase fraction in Sample #5 is consistent with the result determined by SR-PXRD. Figure 3 shows the compared XRD patterns of the as-cast and annealed samples. A small amount of Mg_2_Ni and Mg are still observed in the annealed samples due to the composition of prepared samples deviating from the designed value. On the other hand, it suggests that the solid solution range of Nd_4_Mg_80_Ni_8_ is limited.

After Sample #6 powder is hydrogenated at 350 °C under 2.0 MPa H_2_ for 1 h, the diffraction peaks of Nd_4_Mg_80_Ni_8_ disappear and the pattern could be well indexed by NdH_2.61_, MgH_2_, Mg_2_NiH_0.3_, Mg_2_NiH_4_ and little Mg. Compared the XRD pattern of hydrogenated sample with that of the annealed, it can be seen that the overall amount of Mg_2_Ni and Mg_2_NiH_4_ in hydrogenated sample is obviously larger than that in the annealed one. The evidence suggests that Nd_4_Mg_80_Ni_8_ is decomposed to NdH_2.61_, MgH_2_, Mg_2_NiH_0.3_, Mg_2_NiH_4_ and Mg, because the elements Nd and Mg could only come from Nd_4_Mg_80_Ni_8_. In order to make sure whether NdH_2.61_, MgH_2_ and Mg_2_NiH_4_ are formed *in*-*situ* or not and understand the hydrogen-induced decomposition mechanism of Nd_4_Mg_80_Ni_8_, the microstructure and phase composition of the incompletely hydrogenated bulk Sample #5 was investigated by HR-TEM.

### The mechanism of hydrogen-induced decomposition and the formation of NdH_2.61_-MgH_2_-Mg_2_NiH_0.3_ nanocomposites

**Figure 4:**
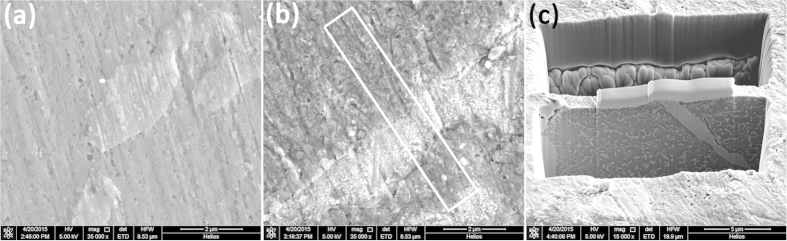
The microstructure of Nd_4_Mg_80_Ni_8_: (a) before hydrogenation, (b) after hydriding at 350 °C for 1 h, and (c) the vertical section of hydrogenated sample.

The comparison of the microstructure of Nd_4_Mg_80_Ni_8_ and incompletely hydrogenated sample is shown in Fig. 4. The starting material shows plate-like Mg_2_Ni in the Nd_4_Mg_80_Ni_8_ matrix. After hydrogenation the surface of Nd_4_Mg_80_Ni_8_ becomes rough. Many ultrafine white particles are observed on the Mg_2_Ni. The original Nd_4_Mg_80_Ni_8_/Mg_2_Ni phase boundaries in the starting alloy are still visible. By excavating holes on the hydrogenated sample, the inner microstructure is shown in Fig. 4(c). Many second phases precipitate from the Nd_4_Mg_80_Ni_8_ matrix after hydrogenation.

**Figure 5:**
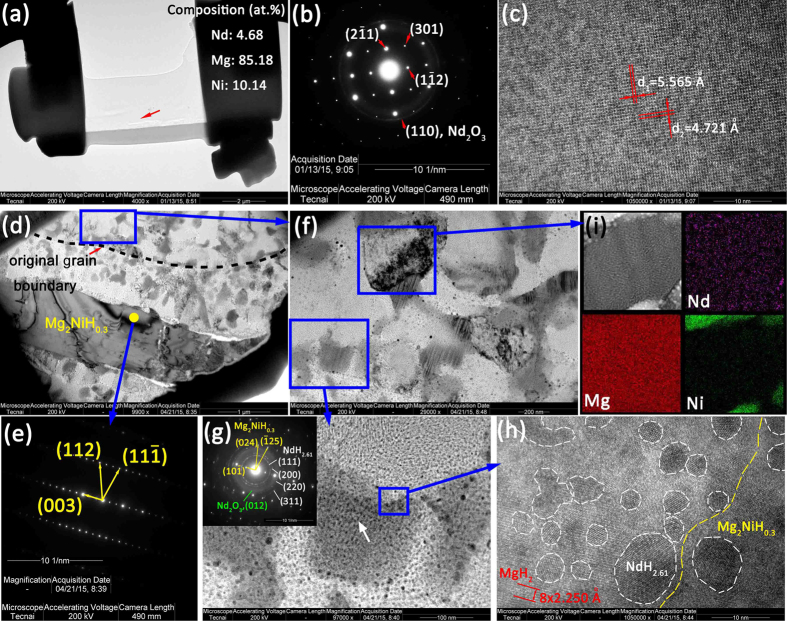
(**a**) The TEM bright-field image of Nd_4_Mg_80_Ni_8_, (**b**) the SAED pattern of Nd_4_Mg_80_Ni_8_, (**c**) HR-TEM image of Nd_4_Mg_80_Ni_8_, (**d**) the bright field image of incompletely hydrogenated Sample #6, (e) SAED pattern of Mg_2_NiH_0.3_, (**f**) the magnified image of the incompletely hydrogenated Nd_4_Mg_80_Ni_8_, (**g**) the magnified image of MgH_2_ and Mg_2_NiH_0.3_ and the SAED pattern of dark phase, (**h**) the HR-TEM image of MgH_2_/Mg_2_NiH_0.3_ interface, and (**i**) the composition map of Nd, Mg and Ni.

HR-TEM was used to determine the phase composition and analyze the microstructure evolution during hydrogenation. The bright-field image, selected area electron diffraction (SAED) pattern and HR-TEM image of the Nd_4_Mg_80_Ni_8_ are shown in Fig. 5 (a~c). The composition of the selected area agrees well with the nominal composition of Nd_4_Mg_80_Ni_8_. The indexing of SAED pattern is consistent with the result of SR-PXRD. In addition, a weak polycrystalline diffraction ring is observed with interplanar spacing *d* = 2.105 Å which corresponds to the crystal plane of (110) of Nd_2_O_3_ (*d* = 1.9735 Å). It indicates that a little Nd_2_O_3_ formed on the surface of the thin slice. From the aspect of thermodynamics, the oxide of Nd should form earlier than the oxide of Mg and Ni because of the lower Gibbs free energy of formation of Nd oxide. The interplanar spacing of *d*_1_ and *d*_2_ in Fig. 5(c) correspond to the crystal planes of 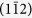 and 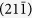 of Nd_4_Mg_80_Ni_8_. Both values agree with 5.6323 Å and 4.8066 Å determined by SR-PXRD with relative error of 1.36% and 1.80%, respectively. The HR-TEM image shows that the Nd_4_Mg_80_Ni_8_ compound is homogeneous and highly crystallized.

Figure 5(d) shows the bright field image of the incompletely hydrogenated sample. The plate-like Mg_2_Ni in starting alloy dissolves little hydrogen to become the solid solution of Mg_2_NiH_0.3_, which is identified by the SAED pattern given in Fig. 5(e). The composition of the Mg_2_NiH_0.3_ at the position marked by yellow point is at.% 0.46Nd-66.05Mg-33.49Ni. The hydrogen cannot be detected from EDS. From Fig. 5(d), an original grain boundary in the starting alloy of Nd_4_Mg_80_Ni_8_ is observed. Many fine equiaxed dark particles with size in the range of 58 ~ 250 nm distributed randomly in the bright matrix.

Figure 5(f) shows the magnified TEM image of the region which was single phase Nd_4_Mg_80_Ni_8_ in starting material. From Fig. 5(g), it can be seen that numerous ultrafine particles distribute in both bright and dark phases. The composition of dark phase is at.% 4.47Nd-71.78Mg-23.76Ni. It is Mg_2_NiH_0.3_ which is identified by SAED pattern shown in Fig. 5(g). The index of the diffraction rings indicates that the high density particles in Mg_2_NiH_0.3_ are NdH_2.61_ nanoparticles. The high content of 4.47 at.% Nd on the Mg_2_NiH_0.3_ is contributed by the NdH_2.61_. Figure 5(h) shows the high resolution TEM image of the region marked as blue frame in Fig. 5(g). The interplanar spacing of the bright matrix is *d* = 2.250 Å which corresponds to the crystal plane (110) of MgH_2_ (*d* = 2.2570 Å). Numerous equiaxed grains of NdH_2.61_ are distributed in the matrix MgH_2_. The average composition of bright region is at.% 5.70Nd-93.23Mg-1.07Ni. The difference of Nd content in Mg_2_NiH_0.3_ and MgH_2_ phases is small, which also indicates that the NdH_2.61_ particles distribute uniformly in both MgH_2_ and Mg_2_NiH_0.3_ phases. The NdH_2.61_ is nanocrystalline with grain size of about 4 ~ 40 nm. Figure 5(i) gives the composition map of Nd, Mg and Ni. It shows that the Nd element concentrates in the nanoparticles and Ni element concentrates in Mg_2_NiH_0.3_ phases.

Based on the TEM results, the mechanism of hydrogen-induced microstructure evolution can be revealed. When the alloy reacts with hydrogen, the Nd atoms firstly disassociate from Nd_4_Mg_80_Ni_8_ to generate NdH_2.61_ because the enthalpy of formation of NdH_2.61_ (−207.2 ~ −187.6 kJ/mol^35^,^36^) is relatively lower than that of MgH_2_ and Mg_2_NiH_4_. After the ultrafine nanoparticles of NdH_2.61_ *in*-*situ* generating from the original Nd_4_Mg_80_Ni_8_ compound, the structure of Nd_4_Mg_80_Ni_8_ becomes unstable because the absence of Nd atoms leads to the polyhedra of [NdMg_16_] crumbling. The released Mg atoms make the structure highly disorder. According to the equilibrated phase diagram, the rest composition will shift to Mg + Mg_2_Ni two-phase region. The Mg and Ni atoms diffuse fast owing to the disordered structure. Then large particles of Mg and Mg_2_Ni (58 ~2 50 nm) form to reduce the Gibbs free energy of system. After the Nd_4_Mg_80_Ni_8_ transforming to NdH_2.61_-Mg-Mg_2_Ni nanocomposites, the Mg reacts with hydrogen to generate MgH_2_ and the hydrogen atoms dissolve in Mg_2_Ni to generate Mg_2_NiH_0.3_. The high density NdH_2.61_ nanoparticles, numerous interfaces between MgH_2_ and Mg_2_NiH_0.3_, and a large number of grain boundaries in the nanocomposites of NdH_2.61_-Mg-Mg_2_Ni may provide a great deal of hydrogen atoms diffusion channels and nucleation sites of hydrides. Thus, the NdH_2.61_-MgH_2_-Mg_2_NiH_0.3_ nanocomposites should exhibit excellent H/D kinetics.

### The thermodynamic and kinetic properties of H/D reactions in Nd_4_Mg_80_Ni_8_

**Figure 6:**
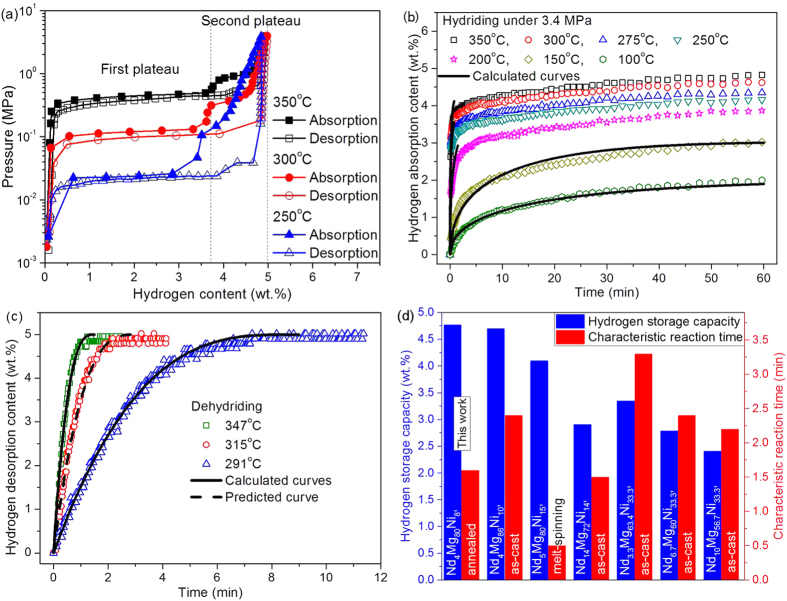
(**a**) The PCT curves of Nd_4_Mg_80_Ni_8_ at 250, 300 and 350 °C, (**b**) the absorption kinetic curves in the temperature range of 100 ~ 350 °C, (**c**) the desorption kinetic curves at 291, 315 and 347 °C, and (**d**) the comparison of hydrogen storage capacity and hydriding rate for Nd-Mg-Ni alloys at 300 °C.

The Sample #6 shows a good activation behavior at 350 °C. At the second H/D cycle, it reaches a maximum hydrogen capacity of 5.15 wt.% which is near to the theoretical value 5.18 wt.% H_2_. All the PCT curves at different temperatures shown in Fig. 6(a) manifests two flat plateaus, indicating that there are two phases reacting with hydrogen during the H/D processes. One of phases exhibits larger storage capacity and wider plateau of H/D reactions marked as the first plateau in Fig. 6(a). The second phase shows higher equilibrium pressure of hydrogen and narrower plateau marked as the second plateau. Table 3 gives plateau pressures, maximum hydrogen capacities at different temperatures and thermodynamic data for the different phases. The hysteresis factor defined as Hf = ln(*P*_ab_/*P*_de_) are 0.144, 0.206 and 0.134 at 350, 300 and 250 °C respectively for the first plateau and 0.474, 0.693 and 1.366 for the second plateau. The enthalpies and entropies are derived from the Van’t Hoff equation. The enthalpies of H/D reactions for the first phase are consistent with the reported values of MgH_2_ in the range of 71.9 ~ 78.0 kJ/mol^8^,^17^,^21^,^37^. The enthalpies of H/D reactions for the second phase are consistent with the reported values of Mg_2_NiH_4_ in the range of 53.23 ~ 72.9 kJ/mol^38^,^39^,^40^,^41^,^42^,^43^. The comparison indicates that both thermodynamic properties of MgH_2_ and Mg_2_NiH_4_ are basically unchanged by adding 4.5 at.% NdH_2.61_.

Figure 6(b) shows the hydriding behavior of Nd_4_Mg_80_Ni_8_ in the temperature range from 100 to 350 °C. It absorbs 85% of the maximum hydrogen content above 250 °C within 5.8 min. After that the hydrogen absorption content increases slowly with prolonging time. At 1 h, the alloy absorbs 4.82 wt.% hydrogen at 350 °C, which is 93% of the theoretical hydrogen storage content. The sample exhibits good desorption kinetics as shown in Fig. 6(c). It releases the absorbed hydrogen thoroughly within 8.3 min when the sample is heated up to 291 °C.

Lots of scholars developed kinetic models for the gas-solid reaction, such as Jander model^44^, Ginstling-Brounshtein equation^45^, etc. Evard *et al.*^46^ developed a mathematical model to describe the non-isothermal decomposition process of MgH_2_, which took into account relative rates of hydrogen desorption, chemical transformation on the MgH_2_-Mg interface and size distribution of the powder particles. In our previous work, Chou *et al.*^47^,^48^,^49^ proposed a series of formulae concerning the isothermal kinetics of gas-solid reaction based on a real physical picture. All parameters in RPP model have clear physical meanings and the effects of temperature, pressure, particle size, sample shape, density change of resultant on the reaction fraction can be analyzed quantitatively. The treatment of this model avoids the multistep calculation error at multi-temperatures and multi-pressures^50^. up to now, the RPP model has been successfully used in analyzing the H/D kinetics of Mg-Ni alloy^50^,^51^,^52^, LaNi_5_-based alloy^9^, Mg-LaNi_5_^53^, La_2_Mg_17_-based composites^54^, etc. Therefore, the isothermal H/D kinetics of the Nd_4_Mg_80_Ni_8_ are analyzed by fitting the observed curves using the RPP model. It is found that the rate controlling step is the diffusion of hydrogen in the hydride during hydrogenation by fitting the experimental data with Eq. (5).


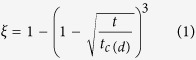


where


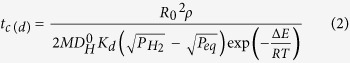


ξ the reacted fraction equaling to the ratio of hydrogen absorption weight Δ*m* at time *t* to the maximum hydrogen absorption weight Δ*m*_max_, *t*_c(d)_ the characteristic reaction time representing the required time of a completely hydriding or dehydriding of the sample, *P*_H2_ the partial pressure of hydrogen in gas phase, *P*_eq_ the hydrogen partial pressure in equilibrium with hydride, and Δ*E* the activation energy. The characteristic time *t*_c_ is regarded as a criterion for reaction rate: the larger the characteristic time, the slower the reaction rate. The corresponding squared correlation coefficient, *r*^2^, reflects the level of agreement between fitting curve and experimental data. Using Eq. (1) to fit the hydrogenation data, the calculated *t*_c(d)_ decreases from 153.5 to 1.6 min when temperature increases from 100 to 300 °C, indicating that the hydriding reaction rate increases with the temperature rising. The apparent activation energy for hydrogenation is determined to be 82.3 kJ/mol by fitting the experimental data using Eq. (2).

There is an interesting phenomenon that the fastest hydriding rate is observed at 300 °C for Nd_4_Mg_80_Ni_8_ (*t*_c(d)_ = 1.6 min) alloy within the investigated temperature range from 100 to 350 °C. It is known that both the forward reaction rate (hydriding reaction) and reverse reaction rate (dehydriding reaction) are accelerated with the increasing temperature. In addition, the hydrogenation of Mg and Mg_2_Ni is exothermic, while the dehydrogenation reaction is endothermic. The increase of temperature is propitious for the reverse reaction. If the reverse reaction rate increases more rapidly than the forward reaction rate, an apparent fastest hydriding rate would be found in the temperature range.

In order to compare the hydriding rate of Nd_4_Mg_80_Ni_8_ with that of other Nd-Mg-Ni alloys in literatures^12^,^13^,^16^,^18^,^26^, the calculated results of characteristic reaction time are listed in Table S2. Figure 6(d) shows the comparison of hydrogen storage capacity and hydriding rate of those alloys at 300 °C. Except the samples prepared by melt-spinning, the fastest two hydrogenation are observed from Nd_14_Mg_72_Ni_14_ (*t*_c(d)_ = 1.5 min) and Nd_4_Mg_80_Ni_8_ (*t*_c(d)_ = 1.6 min) alloys at 300 °C. However, the maximum hydrogen absorption content of Nd_14_Mg_72_Ni_14_ is only 2.91 wt.%, relatively smaller than that of Nd_4_Mg_80_Ni_8_ (4.77 wt.%) at the same temperature. The composition of as-cast Nd_4_Mg_86_Ni_10_ alloy^13^ is very close to Nd_4_Mg_80_Ni_8_ developed in present work, but the as-cast Nd_4_Mg_86_Ni_10_ is a multiphase alloy consisted of NdMg_12_, Mg and Mg_2_Ni phases. This indicates that the composition distribution in the annealed Nd_4_Mg_80_Ni_8_ is more uniform than that in as-cast Nd_4_Mg_86_Ni_10_, which results in that the *in*-*situ* formed NdH_2.61_-Mg-Mg_2_Ni nanocomposites more homogenous than that in as-cast Nd_4_Mg_86_Ni_10_. As calculated from RPP model, the characteristic hydriding time at 300 °C for Nd_5_Mg_80_Ni_15_ (12, 3.5 MPa), Nd_4_Mg_80_Ni_8_ (this work, 3.4 MPa) and Nd_4_Mg_86_Ni_10_ (13, 3.0 MPa) are 0.5, 1.6 and 4.7 min, respectively. It indicated that the hydriding rate of Nd_5_Mg_80_Ni_15_ alloy is the fastest, while the hydriding rate of Nd_4_Mg_86_Ni_10_ is much slower than that of the other two alloys. The hydriding rate is related to many factors, such as temperature, hydrogen pressure, microstructure, powder size, composition, etc. Both of Nd_5_Mg_80_Ni_15_ and Nd_4_Mg_80_Ni_8_ using for hydriding are nanocrystalline structure, but the particle size of Nd_5_Mg_80_Ni_15_ is much smaller than the micro-particles of Nd_4_Mg_80_Ni_8_ (−100 mesh) and Nd_4_Mg_86_Ni_10_ (~70 mesh). The smaller the sample size, the faster the hydriding rate. In addition, the Ni content in Nd_5_Mg_80_Ni_15_ is higher than that in Nd_4_Mg_80_Ni_8_ and Nd_4_Mg_86_Ni_10_, which means the quantity of catalytic element is more than Nd_4_Mg_80_Ni_8_ and Nd_4_Mg_86_Ni_10_. Therefore, the hydriding rate of melt-spinning and crystallized Nd_5_Mg_80_Ni_15_ showed better hydriding kinetics than that of Nd_4_Mg_80_Ni_8_ and Nd_4_Mg_86_Ni_10_. But the increase of Ni content reduced the hydrogen storage capacity (Nd_5_Mg_80_Ni_15_: 4.10wt.%, Nd_4_Mg_80_Ni_8_: 4.77wt.%, and Nd_4_Mg_86_Ni_10_: 4.70wt.%). The comparison of all alloys in Fig. 6(d) shows that the optimal Nd-Mg-Ni alloy is the Nd_4_Mg_80_Ni_8_ designed in present work by considering the hydriding kinetics and hydrogen storage capacity.

The rate controlling steps are surface penetration (sp) of hydrogen atoms for dehydriding reaction at 291 ~ 347 °C through fitting the experimental data with Eq. (7).


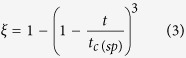


where


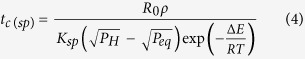


The calculated *t*_c(sp)_ are 8.8, 3.0 and 1.5 min with temperature increasing from 291 to 347 °C. This means that the dehydriding reaction rate increases with the temperature rising. The activation energy for dehydrogenation is calculated to be 97.5 kJ/mol, which is much smaller than 160 kJ/mol for ball milled pure MgH_2_^55^, 124.6 kJ/mol for induction melted Mg_90_Ce_5_Ni_5_ alloy^8^, and comparable to 104 kJ/mol for the as-cast CeMg_3_^21^. Combining Eqs (3 and 4), the dehydriding kinetic curves at any other temperatures can be predicted by RPP model as follows:


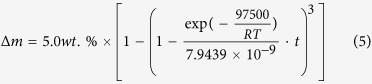


where *R* the gas constant, *T* temperature in Kelvin, and 5.0 wt.% the largest desorption hydrogen content from experimental. The calculated and predicted curves are shown in Fig. 6(c), which suggests that the theoretical calculation agree well with experimental data.

**Figure 7:**
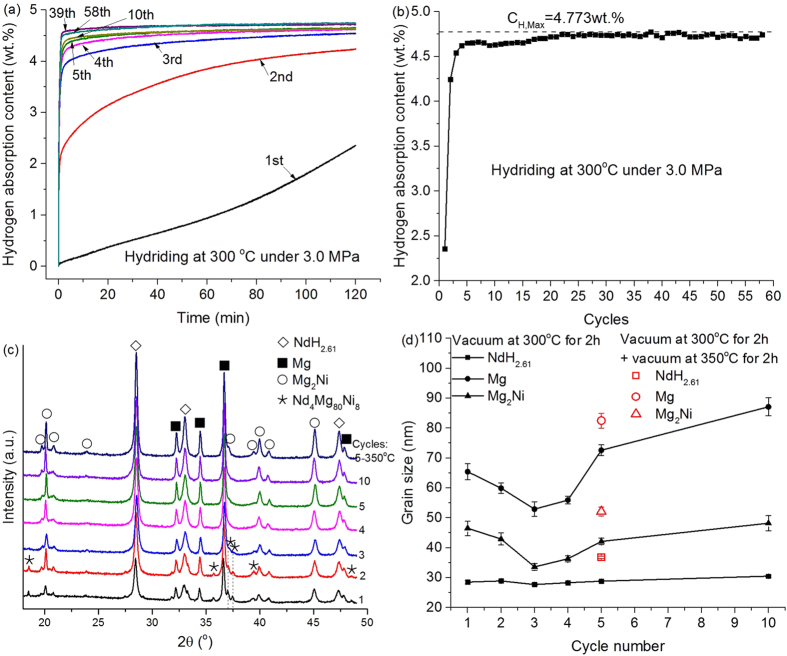
(**a**) The hydriding behavior of Nd_4_Mg_80_Ni_8_ at 300 °C under 3.0 MPa H_2_ at different cycles, (**b**) the hydrogen storage capacity versus cycle times, (**c**) the XRD patterns of completely dehydrogenated samples after different cycles, and (**d**) the relationship between grain size and cycle number.

The cycle life kinetics was examined at 300 °C under 3.0 MPa H_2_. The hydriding behaviors of the 1^st^ ~ 5^th^, 10^th^, 39^th^ and 58^th^ cycles are showed in Fig. 7(a). It can be seen that the hydriding rate increases with the increase of cycle times from 1^st^ to 5^th^. After the 5^th^ cycle, the hydriding rate becomes very fast. Figure 7(b) shows the hydrogen storage capacity versus cycle times. The hydrogen capacity increases sharply from 2.36 to 4.54 wt.% in the first 3 cycles and then increases gradually to the maximum value of 4.77 wt.%. Until the 58^th^ cycle, the hydrogen storage capacity still remains stable, which suggests that the Nd_4_Mg_80_Ni_8_ has a good cycle ability. In order to investigate the relationship between grain size and cycle times, the XRD pattern was collected after the 1^st^ ~ 5^th^ and 10^th^ cycles, shown in Fig. 7(c). The samples were vacuumed at 300 °C for 2 h. A part of powders after the 5^th^ cycle were further vacuumed at 350 °C for 2 h.

The calculated grain size versus cycle numbers is showed in Fig. 7(d). It can be seen that the grain size of NdH_2.61_ increase slowly with the cycle number, but the grain sizes of Mg and Mg_2_Ni decrease in the first 3 cycles and then increase with the increase of cycle number. The Nd_4_Mg_80_Ni_8_ disappears until the 4^th^ cycle and the phase fractions of Nd_4_Mg_80_Ni_8_ in the first 3 cycles are 7.0 ± 0.6, 4.1 ± 0.2 and 2.5 ± 0.5 wt.%, respectively. Although most of Nd_4_Mg_80_Ni_8_ decomposed in the 1^st^ cycle, the hydrogen absorption content is only 2.36wt.%. It suggests the generated Mg and Mg_2_Ni didn’t absorb hydrogen fully. Therefore, in the 2^nd^ and 3^rd^ cycle, the uncompleted phase transformation of Mg ↔ MgH_2_ and Mg_2_Ni ↔ Mg_2_NiH_4_ reduced the grain size of Mg and Mg_2_Ni. After the sample is completely activated, the phase transformation can be finished at the initial stage of H/D process. The long holding time at this temperature leads to the growth of grain size. Therefore, after the 4^th^ cycle the grain sizes of Mg and Mg_2_Ni growth obviously.

The grain size of the sample further vacuumed at 350 °C is larger than that dehydriding at 300 °C. It suggests that the grain size grows with the raise of temperature and the extension of time. The growth of NdH_2.61_ is slowly with cycle times, but the growth of Mg is obviously from 60 ± 2 nm after the 3^rd^ cycle to 87 ± 3 nm after the 10^th^ cycle. The grain size of Mg vacuumed at 350 °C is about 83 ± 3 nm which is smaller than the value of Mg (150 nm) reported by Denys *et al.*^20^ at the same temperature. This is because it was pure Mg sample used in their study, while the well-distributed NdH_2.61_ and Mg_2_Ni in present work can restrain the growth of Mg^21^.

### The phase transformation of NdH_2.61_-Mg-Mg_2_Ni nanocomposites during hydrogenation

**Figure 8:**
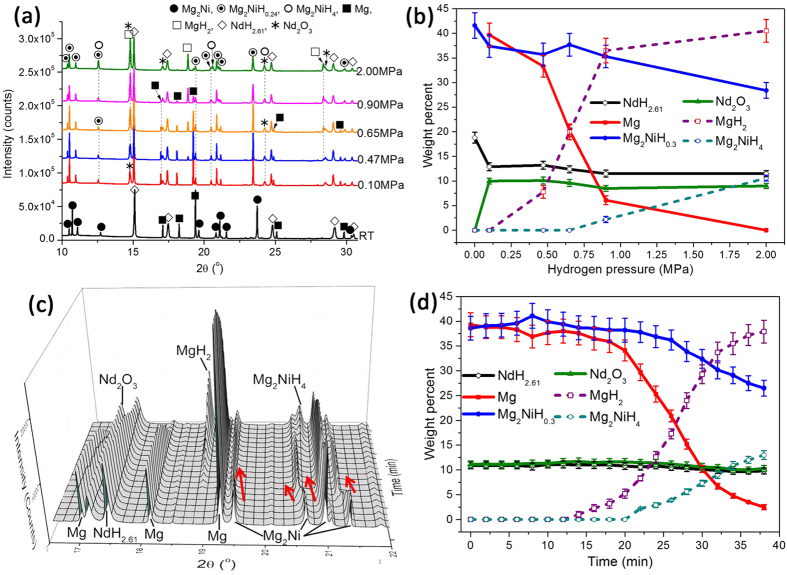
(**a**) The SR-PXRD patterns of NdH_2.61_-Mg-Mg_2_Ni composites under different hydrogen pressures at 350 °C, (**b**) the weight fraction of each phase versus pressure, (**c**) the SR-PXRD patterns of NdH_2.61_-Mg-Mg_2_Ni composites during hydriding process at different time at 300 °C, and (**d**) the weight fraction of each phase versus time.

The phase evolution mechanism of RE-Mg-Ni alloys during hydrogenation/dehydrogenation process was well clarified by Denys *et al.*^20^,^24^,^25^ combining *in-situ* SR-PXRD. The effect of solidification rate on the microstructure of alloy, phase structural and microstructural state of constituents during reversible process of synthesis and decomposition of hydrides, and kinetic mechanism during hydriding and dehydriding process were studied in detail. Inspiring by their work, the *in*-*situ* SR-PXRD assisted with Rietveld refinement was also applied to study the mechanism of phase transformation under different hydrogen pressures and at different time. The SR-PXRD patterns under different hydrogen pressures at 350 °C are shown in Fig. 8(a). The indexation of the pattern of the activated powders indicates the existence of NdH_2.61_, MgH_2_ and Mg_2_Ni. Combined with the TEM results, the reaction taking place during the first hydrogenation is assumed to be:


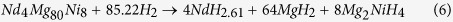


The calculated fraction of each phase versus pressure is plotted in Fig. 8(b). The sample of complete dehydrogenation contained 18.8 wt.% NdH_2.61_, 39.5 wt.% Mg and remainder Mg_2_Ni. As temperature increased to 350 °C under 0.10 MPa H_2_, the phase fraction of Mg and Mg_2_Ni didn’t change. However, 10.0 wt.% Nd_2_O_3_ emerged because of the oxidation of NdH_2.61_ during heating process. Then the content of Nd_2_O_3_ remained stable at this level under hydrogen atmosphere. 0.47 MPa H_2_ is close to the pressure of the lower flat plateau at 350 °C. It is found that 7.8 wt.% MgH_2_ appeared and the fraction of Mg reduced to 33.3 wt.%. The result indicates that the flat plateau at 0.47 MPa H_2_ corresponds to the phase equilibrium of Mg + MgH_2_ + NdH_2.61_ + Mg_2_NiH_0.3_. The hydrogen absorption content of 0.15 wt.% H_2_ before the lower flat plateau is contributed by the solid solution of hydrogen in Mg and Mg_2_NiH_0.3_. When the Mg transforms to MgH_2_ entirely, the hydrogen absorption content reaches to 3.74 wt.% (As shown in Fig.6 (a)). The Mg_2_NiH_4_ doesn’t emerge until hydrogen pressure increases to 0.90 MPa. The second flat plateau corresponds to the equilibrium of Mg_2_NiH_0.3_ + Mg_2_NiH_4_ + MgH_2_ + NdH_2.61_. At 2.00 MPa H_2_, the Mg is depleted, but 28.4 wt.% Mg_2_NiH_0.3_ (the solid solution of hydrogen) is remained because of the alloy doesn’t reach the equilibrium state during the measurement. Based on those results, the sequence of the phase transformations with equilibrium hydrogen pressure during hydrogenation at 350 °C is presented as follows:


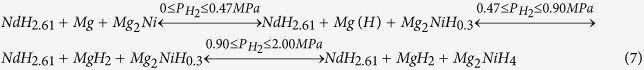


Seen from the isothermal hydriding kinetic curves as shown in Fig. 6(b), the hydriding process of the alloy above 200 °C can be separated as two stages. The first was the rapid hydriding stage, while the second stage exhibited relatively slow hydriding rate. The phase composition during the hydriding process at 300 °C under 2.00 MPa H_2_ was analyzed by *in*-*situ* SR-PXRD, shown in Fig. 8(c). The Mg_2_Ni phase peaks shifts left toward lower 2θ values as time increases to 8 min (marked as the red arrows) which suggests the lattice expansion of the Mg_2_Ni phase causing by the solid solution of hydrogen atoms. The intensity of Mg and Mg_2_Ni decreases with time prolonging, indicating the Mg and Mg_2_Ni transforms to MgH_2_ and Mg_2_NiH_4_, respectively. The change of phase fractions refined by Rietveld method is plotted in Fig. 8(d). About 11.3 wt.% Nd_2_O_3_ emerges and doesn’t change significantly later. There is no hydride appearing until 14 min. The delay of the hydrogenation may be due to the slight oxidation of the powders during heating process. The MgH_2_ appeared earlier than Mg_2_NiH_4_ and its fraction increases with the prolongation of time. At 40 min, the Mg transforms into MgH_2_ almost completely, but 26.5 wt.% Mg_2_NiH_0.3_ is left. The hydrogen absorption process of Mg_8_Mm_20_Ni alloy identified by Denys *et al.*^20^ was as following sequence: (1) Mg_2_Ni → Mg_2_NiH_0.3_, (2) MmH_2_ → MmH_3_ and Mg → MgH_2_, and (3) Mg_2_NiH_0.3_ → Mg_2_NiH_4_. They thought that the transformation of Mg_2_Ni → Mg_2_NiH_0.3_ occurred earlier than other transformations is because the instantly formed α-solid solution Mg_2_NiH_0.3_ catalyzed the hydrogenation of Mg. Therefore, one can believe that the fast hydriding rate of the first stage results from the fast hydrogenation of Mg.

In summary, the isothermal section of Nd-Mg-Ni system in the Mg-rich corner at 400 °C was established based on the phase relationships determined from equilibrated alloys. A new ternary compound Nd_4_Mg_80_Ni_8_ was found and it exhibited excellent H/D kinetic properties as a novel hydrogen storage alloy. It has structure of space group *I*4_1_/*amd* (No.142), Z = 4, *a = b* = 11.2743(1) Å, *c* = 15.9170(2) Å. HR-TEM results revealed that the hydrogen-induced decomposition of Nd_4_Mg_80_Ni_8_ lead to *in*-*situ* formation of NdH_2.61_-MgH_2_-Mg_2_NiH_0.3_ nanocomposites. The high density grain boundaries in the nanocomposites of NdH_2.61_-Mg-Mg_2_Ni provided a great deal of hydrogen atoms diffusion channels and nucleation sites of hydrides, which greatly enhances the H/D kinetics and improved the cycle ability. The grain size of NdH_2.61_ grows slowly with cycle number, but the grain sizes of Mg and Mg_2_Ni decrease in the first 3 cycles, and then increase with the increase of cycle times. The growth of grain size is related with temperature and vacuum time. The kinetics mechanism is analyzed by RPP model, which suggests that the rate controlling step was diffusion for hydrogenation and surface penetration for dehydrogenation. The *in*-*situ* SR-PXRD results revealed that the sequence of phase transformation during hydrogenation at 350 °C was NdH_2.61_ + Mg + Mg_2_Ni → NdH_2.61_ + Mg(H) + Mg_2_NiH_0.3_ → NdH_2.61_ + MgH_2_ + Mg_2_NiH_0.3_ → NdH_2.61_ + MgH_2_ + Mg_2_NiH_4_ with the increase of equilibrium hydrogen pressure from 0.0 to 2.0 MPa. Mg absorbed hydrogen earlier and faster than Mg_2_Ni during isothermal hydrogenation at 300 °C under 2.0 MPa H_2_.

## Experimental Methods

### The preparation and examination of the equilibrated alloys

The Nd-Mg-Ni samples were prepared by a medium frequency induction furnace using blocks of Nd (≥99.99 wt.%), Mg (≥99.99 wt.%) and Ni (≥99.99 wt.%) as the starting materials. The as-cast samples were enclosed by tantalum foils for subsequently sealing in evacuated quartz tubes. The samples were annealed at 400 °C for 30 days and then quenched in ice-water. The sample compositions and heat treatment conditions were listed in Table S1.

The actual composition of each alloy was determined by inductively coupled plasma atomic emission spectrometry (ICP). The microstructure and composition of phases in the bulk samples were investigated by HITACHI SU-1500 scanning electron microscopy (SEM) equipped with energy dispersive X-ray spectrometer (EDS). The phase composition of annealed samples were characterized by X-ray diffraction (XRD) using 18KW D/MAX2500V + /PC diffractometer with Cu Kα radiation.

### The solution of crystal structure

According to the average composition of at.% 4.86Nd-87.46Mg-7.68Ni detected from EDS, the Nd_4_Mg_80_Ni_8_ compound (Sample #5) was prepared by annealing an induction melted ingot at 400 °C for 30 days followed by ice-water quenching. The actual composition determined by ICP located in the region of Nd_4_Mg_80_Ni_8_ + Mg two-phase equilibrium. SR-PXRD data for Sample #5 were collected at a wavelength of 0.8262 Å by a Mythen-II detector on powder diffraction beamline, Australian synchrotron. The powdered samples were loaded into pre-dried 0.7 mm quartz capillaries fitted with a flow cell under an atmosphere of argon. The Rietveld refinement was performed using TOPAS v4.2^32^.

### The microstructure of Nd_4_Mg_80_Ni_8_ and its hydrogen-induced microstructure evolution

A bulk sample of Nd_4_Mg_80_Ni_8_ compound (Sample #6, actual composition is Nd_4.5_Mg_84.6_Ni_10.9_) with size of 3 × 3 × 2 mm was polished to obtain a smooth surface. The sample was consist of major phase Nd_4_Mg_80_Ni_8_ and minor Mg_2_Ni. Then the bulk sample was incompletely hydrogenated at 350 °C under 2.0 MPa H_2_ for 1 h. The microstructure of the sample was examined by FEI Helios Nanolab 600i dual beam focused ion beam FIB. In order to compare the microstructure and phase composition of Nd_4_Mg_80_Ni_8_ and hydrogenated sample, two thin slices with thickness less than 100 nm were cut from the annealed Nd_4_Mg_80_Ni_8_ and hydrogenated sample respectively by FEI Helios Nanolab 600i dual beam FIB. SAED patterns and HR-TEM images were collected by Tecnai G2 F20 S-Twin TEM.

### The measurement of H/D properties

The H/D properties of annealed Nd_4_Mg_80_Ni_8_ (Sample #6) was tested using automatic PCT characteristics measurement system from SUZUKI HOKAN. CO., LTD. in Japan. The Nd_4_Mg_80_Ni_8_ was mechanically crushed into micro-particles (−100 mesh, <150 μm) and activated at 350 °C under 4.0 MPa H_2_ for hydrogen absorption and at the same temperature in vacuum for hydrogen desorption. The PCT curves were measured at 250 ~ 350 °C with the maximum equilibrated time of 40 min. The hydrogen absorption kinetics were examined at 100 ~ 350 °C under initial hydrogen pressure of 3.4 MPa. Before hydrogenation the sample was kept in vacuum at 350 °C for 2 h to ensure its complete dehydrogenation. The isothermal dehydriding kinetics was examined at 291 ~ 347 °C in vacuum after the sample completely hydriding at 350 °C for 2 h under initial hydrogen pressure of 3.4 MPa. The cycling behavior of the Nd_4_Mg_80_Ni_8_ was determined at 300 °C. The time for hydriding under initial pressure of 3.0 MPa H_2_ was 2 h and for dehydriding in vacuum was 2.8 h. In order to study the relationship between grain size and cycle number, the XRD pattern was collected after the 1^st^ ~ 5^th^ and 10^th^ cycles. The samples were vacuumed at 300 °C for 2 h and then air cooled to room temperature. A part of powders after the 5^th^ cycle were further vacuumed at 350 °C for 2 h to observe the growth of grain size. The XRD patterns were collected by Bruker AXS D8 diffractometer with Cu Kα radiation. The sizes of the crystallites in the samples were calculated from the refinements of XRD patterns using Scherrer equation.

### The evolution of phase composition during hydrogenation

The *in-situ* SR-PXRD data of Nd_4.5_Mg_84.6_Ni_10.9_ powders were collected at by a wavelength of 0.8262 Å by a Mythen-II detector on powder diffraction beamline, Australian synchrotron. The completely dehydrogenated Nd_4.5_Mg_84.6_Ni_10.9_ powders were loaded into pre-dried 0.7 mm quartz capillaries fitted with a flow cell under an atmosphere of argon. The sample was heating to the set temperatures by a Cybostar hot air blower with heating rate of 20 °C/min under vacuum, then hydrogen was imported in and the detector started to collect the XRD data. Before heating, the quartz capillaries didn’t scrubbing with argon, which led to some oxidation of the sample during heating process. Therefore, about 10.0 wt.% Nd_2_O_3_ emerged at the initial stage of examination. Then the quantity of Nd_2_O_3_ didn’t change any more. The phase transformation of Mg and Mg_2_Ni during the hydriding process still can be observed. The diffraction data under different hydrogen pressures from 0.0 to 2.0 MPa were collected at 350 °C and proceeded for 4 min at one pressure. Before every collection, the sample was kept under this pressure for 30 min. The diffraction data at different time was measured every 2 min at 300 °C under a constant pressure of 2.0 MPa H_2_.

## Additional Information

**How to cite this article**: Luo, Q. *et al.* Phase Equilibria, Crystal Structure and Hydriding/Dehydriding Mechanism of Nd_4_Mg_80_Ni_8_ Compound. *Sci. Rep.* **5**, 15385; doi: 10.1038/srep15385 (2015).

## Supplementary Material

Supplementary Information

## Figures and Tables

**Table 1:** Crystallographic data and structure refinement of Nd_4_Mg_80_Ni_8._

Formula sum	Nd_4_Mg_80_Ni_8_
Formula weight	2990.88 g/mol
Crystal system	Tetragonal
Space group	*I* 4_1_*/a m d* (No. 141)
Cell parameters	*a* *=* *b* = 11.2743(1) Å, *c* = 15.9170(2) Å
Cell volume	2023.19(4) Å^3^
Z	4
Calc. density	2.45462 g/cm^3^
*R*_wp_	8.1%
*R*_B_	6.4%
*Go*F	1.44

**Table 2:** The atomic parameters of Nd_4_Mg_80_Ni_8_.

Atoms	Wyck.	S.O.F	*x*/*a*	*y*/*b*	*z*/*c*	B iso (Å^2^)
Mg1	16*h*	1	0.13260	1/2	0.54657	1.865
Mg2	8*d*	1	0	1/4	5/8	1.865
Mg3	8*e*	1	1/2	1/2	5/8	1.865
Mg4	32*i*	1	0.36417	0.26792	0.31625	1.865
Mg5	16*g*	1	0.36383	0.63617	1/2	1.865
Ni1	8*c*	1	0	1/4	1/8	1.960
Nd1	4*b*	1	0	0	1/2	1.553

**Table 3:** The H/D properties and thermodynamic data of Nd_4_Mg_80_Ni_8._

Plateau	*T* (°C)	*P*_ab_ (MPa)	*P*_de_ (MPa)	*C*_ab_ (wt.%)	Calculated Δ*H* (kJ/mol) and Δ*S* (J/(mol·K))	
					Absorption	Desorption
First plateau (0 ~ 3.74wt.%H)	350	0.441	0.382	3.74	Δ*H* = −79.1 ± 0.5	Δ*H* = 784 ± 1.9
	300	0.118	0.096	3.62	Δ*S* = −120.3 ± 0.8	Δ*S* = 117.6 ± 3.3
	250	0.024	0.021	3.41		
Second plateau (3.74 ~4 .94wt.%H)	350	0.908	0.565	1.20	Δ*H* = −48.8 ± 2.5	Δ*H* = 727 ± 3.2
	300	0.368	0.184	1.36	Δ*S* = −77.3 ± 4.3	Δ*S* = 112.3 ± 5.6
	250	0.149	0.038	1.43		

Note: *P*_ab_ and *P*_de_ are H/D plateau pressures, *C*_ab_ is capacities of hydrogen absorption.
