# Supplementary material for: Phase Equilibria, Crystal Structure and Hydriding/Dehydriding Mechanism of Nd4Mg80Ni8 Compound
Source: Sci Rep. 2015 Oct 16;5:15385. doi: 10.1038/srep15385 (PMC4607946; doi:10.1038/srep15385)
Supplement: Supplementary Information [file srep15385-s1.doc]

**Phase Equilibria, Crystal Structure and Hydriding/Dehydriding Mechanism of Nd_4_Mg_80_Ni_8_ Compound**

**Supplementary Information**

Qun Luo ^a^, Qin-Fen Gu ^b^, Jie-Yu Zhang ^a^, Shuang-Lin Chen ^a, d^, Kuo-Chih Chou ^a^, Qian Li ^a,c^,*

*^a^ State Key Laboratory of Advanced Special Steels, Shanghai University, Shanghai 200072, China*

*^b^ Australian Synchrotron, 800 Blackburn Rd, Clayton 3168, Australia*

*^c^ Institute of Genomic Material, Shanghai University, Shanghai 200444, China*

*^d^ CompuTherm, LLC, Madison, WI 53719, USA*

*E-mail: [shuliqian@shu.edu.cn](mailto:shuliqian@shu.edu.cn)

**Supplementary Tables**

Table S1. The phase equilibria data of Nd-Mg-Ni system at 400 °C

| Samples  No. | Composition (at.%) | Treatments | XRD | SEM-EDS | | | |
| --- | --- | --- | --- | --- | --- | --- | --- |
|  |  |  |  | Phases | Nd | Mg | Ni |
| #1 | Nd 6.67 | 400 °C, 30 days | Nd_5_Mg_41_ | Nd_5_Mg_41_ | 12.20 | 87.33 | 0.47 |
|  | Mg 90.58 |  | Nd_4_Mg_80_Ni_8_ | Nd_4_Mg_80_Ni_8_ | 6.32 | 87.96 | 5.72 |
|  | Ni 2.75 |  | Mg | Mg | 0.27 | 99.72 | 0.01 |
| #2 | Nd 1.53 | 400 °C, 30 days | Nd_4_Mg_80_Ni_8_ | Nd_4_Mg_80_Ni_8_ | 4.29 | 87.25 | 8.46 |
|  | Mg 88.46 |  | Mg | Mg | 0.10 | 99.26 | 0.64 |
|  | Ni 10.01 |  | Mg_2_Ni | Mg_2_Ni | 0.66 | 67.42 | 31.92 |
| #3 | Nd 5.41 | 400 °C, 30 days | NdMg_8_Ni | NdMg_8_Ni | 11.94 | 79.09 | 8.97 |
|  | Mg 80.73 |  | Nd_4_Mg_80_Ni_8_ | Nd_4_Mg_80_Ni_8_ | 4.79 | 86.58 | 8.63 |
|  | Ni 13.86 |  | Mg_2_Ni | Mg_2_Ni | 2.21 | 63.35 | 34.44 |
| #4 | Nd 8.47 | 400 °C, 30 days | NdMg_8_Ni | NdMg_8_Ni | 11.78 | 79.42 | 8.80 |
|  | Mg 85.37 |  | Nd_4_Mg_80_Ni_8_ | Nd_4_Mg_80_Ni_8_ | 4.88 | 87.29 | 7.83 |
|  | Ni 6.16 |  | Nd_5_Mg_41_ | Nd_5_Mg_41_ | 11.48 | 87.85 | 0.67 |

Table S2. The maximum hydrogen absorption content and calculated characteristic time of Nd_4.5_Mg_84.6_Ni_10.9_ alloy compared with that calculated from literature

| Composition | Preparation | Max. W_H_  (wt.%) | Initial *P*  (MPa) | *T*  (°C) | *t*_c(d)_  (min) | *r*^2^ | Ref. |
| --- | --- | --- | --- | --- | --- | --- | --- |
| Nd_4_Mg_80_Ni_8_ | Annealed at 400 °C | 4.82 | 3.4 | 350 | 2.3 | 0.9484 (0≤ξ≤0.87) | This work |
|  |  | 4.62 |  | 300 | 1.6 | 0.9232 (0≤ξ≤0.87) |  |
|  |  | 4.35 |  | 275 | 1.7 | 0.9105 (0≤ξ≤0.80) |  |
|  |  | 4.16 |  | 250 | 1.8 | 0.9159 (0≤ξ≤0.77) |  |
|  |  | 3.87 |  | 200 | 7.0 | 0.9162 (0≤ξ≤0.63) |  |
|  |  | 3.02 |  | 150 | 88.0 | 0.9759 (0≤ξ≤1.00) |  |
|  |  | 2.00 |  | 100 | 153.5 | 0.9882 (0≤ξ≤0.87) |  |
| Nd_14_Mg_72_Ni_14_ | As-cast | 2.91 | 1.0 | 300 | 1.5 | 0.9528 (0≤ξ≤0.95) | [^1^](#_ENREF_1) |
|  |  | 2.73 |  | 200 | 5.1 | 0.9479 (0≤ξ≤0.97) |  |
|  |  | 2.22 |  | 100 | 21.5 | 0.9338 (0≤ξ≤1.00) |  |
| Nd_8.3_Mg_83.4_Ni_8.3_ | Melt-spinning | 4.11 | 3.0 | 150 | 2.2 | 0.9515 (0≤ξ≤0.95) | [^2^](#_ENREF_2) |
| Nd_3.3_Mg_63.4_Ni_33.3_ | As-cast | 3.37 | 4.0 | 350 | 6.4 | 0.9861 (0≤ξ≤0.96) | [^3^](#_ENREF_3) |
|  |  | 3.35 |  | 300 | 3.3 | 0.9912 (0≤ξ≤0.99) |  |
| Nd_6.7_Mg_60.0_Ni_33.3_ | As-cast | 2.88 | 4.0 | 350 | 5.8 | 0.9821 (0≤ξ≤0.98) | [^3^](#_ENREF_3) |
|  |  | 2.79 |  | 300 | 2.4 | 0.9903 (0≤ξ≤0.97) |  |
| Nd_10.0_Mg_56.7_Ni_33.3_ | As-cast | 2.48 | 4.0 | 350 | 3.9 | 0.9997 (0≤ξ≤1.00) | [^3^](#_ENREF_3) |
|  |  | 2.41 |  | 300 | 2.2 | 0.9910 (0≤ξ≤0.98) |  |
| Nd_4_Mg_86_Ni_10_ | As-cast | 4.01 | 3.0 | 350 | 3.1 | 0.8379 (0≤ξ≤0.51) | [^4^](#_ENREF_4) |
|  |  | 4.70 |  | 300 | 2.4 | 0.9306 (0≤ξ≤0.87) |  |
|  |  | 4.75 |  | 250 | 2.3 | 0.9341 (0≤ξ≤0.86) |  |
|  |  | 4.46 |  | 200 | 2.9 | 0.5091 (0≤ξ≤0.93) |  |
|  |  | 4.02 |  | 150 | 7.9 | 0.7057 (0≤ξ≤0.90) |  |
|  |  | 3.42 |  | 100 | 99.0 | 0.9914 (0≤ξ≤1.00) |  |
| Nd_5_Mg_80_Ni_15_ | Melt-spinning, crystallized | 4.10 | 3.5 | 300 | 0.5 | 0.9300 (0≤ξ≤0.88) | [^5^](#_ENREF_5) |

**Supplementary data: CIF file of Nd_4_Mg_80_Ni_8_**

data_

_chemical_name_mineral ?Mg80Ni8Nd4?

_cell_length_a 11.274262(79)

_cell_length_b 11.274262(79)

_cell_length_c 15.91699(23)

_cell_angle_alpha 90

_cell_angle_beta 90

_cell_angle_gamma 90

_cell_volume 2023.192(41)

_symmetry_space_group_name_H-M I41/amd

loop_

_symmetry_equiv_pos_as_xyz

'-x, -y, z'

'-x, -y+1/2, -z+1/4'

'-x, y, z'

'-x, y+1/2, -z+1/4'

'-x+1/2, -y, -z-1/4'

'-x+1/2, -y+1/2, z+1/2'

'-x+1/2, y, -z-1/4'

'-x+1/2, y+1/2, z+1/2'

'-y, -x, -z'

'-y, -x+1/2, z+1/4'

'-y, x, -z'

'-y, x+1/2, z+1/4'

'-y+1/2, -x, z-1/4'

'-y+1/2, -x+1/2, -z+1/2'

'-y+1/2, x, z-1/4'

'-y+1/2, x+1/2, -z+1/2'

'y, -x, -z'

'y, -x+1/2, z+1/4'

'y, x, -z'

'y, x+1/2, z+1/4'

'y+1/2, -x, z-1/4'

'y+1/2, -x+1/2, -z+1/2'

'y+1/2, x, z-1/4'

'y+1/2, x+1/2, -z+1/2'

'x, -y, z'

'x, -y+1/2, -z+1/4'

'x, y, z'

'x, y+1/2, -z+1/4'

'x+1/2, -y, -z-1/4'

'x+1/2, -y+1/2, z+1/2'

'x+1/2, y, -z-1/4'

'x+1/2, y+1/2, z+1/2'

loop_

_atom_site_label

_atom_site_type_symbol

_atom_site_symmetry_multiplicity

_atom_site_fract_x

_atom_site_fract_y

_atom_site_fract_z

_atom_site_occupancy

_atom_site_B_iso_or_equiv

Mg1 Mg 16 0.1326(13) 0.5 0.54657(53) 1 1.865(25)

Mg2 Mg 8 0 0.25 0.625 1 1.865(25)

Mg3 Mg 8 0.5 0.5 0.625 1 1.865(25)

Mg4 Mg 32 0.36417(71) 0.26792(67) 0.31625(44) 1 1.865(25)

Mg5 Mg 16 0.36383(21) 0.63617(21) 0.5 1 1.865(25)

Ni1 Ni 8 0 0.25 0.125 1 1.960(29)

Nd1 Nd 4 0 0 0.5 1 1.553(17)

References

1. Ourane, B. et al., The new ternary intermetallic NdNiMg_5_: Hydrogen sorption properties and more, *Mater. Res. Bull.* **61**, 275-279, (2015).

2. Zhang, Q. A., Jiang, C. J. & Liu, D. D., Comparative investigation on the hydrogenation characteristics and hydrogen storage kinetics of melt-spun Mg_10_NiR (R=La, Nd and Sm) alloys, *Int. J. Hydrogen Energy* **37**, 10709-10714, (2012).

3. Xie, D. H., Li, P., Zeng, C. X., Sun, J. W. & Qu, X. H., Effect of substitution of Nd for Mg on the hydrogen storage properties of Mg_2_Ni alloy, *J. Alloys Compd.* **478**, 96-102, (2009).

4. Yin, J. T., Yamada, T., Yoshinari, O. & Tanaka, K., Improvement of hydrogen storage properties of Mg-Ni alloys by rare-earth addition, *Mater. Trans.* **42**, 712-716, (2001).

5. Tanaka, K. et al., Improvement of hydrogen storage properties of melt-spun Mg-Ni-RE alloys by nanocrystallization, *J. Alloys Compd.* **293-295**, 521-525, (1999).
